# WIPI proteins: Biological functions and related syndromes

**DOI:** 10.3389/fnmol.2022.1011918

**Published:** 2022-09-09

**Authors:** Mohammed Almannai, Dana Marafi, Ayman W. El-Hattab

**Affiliations:** ^1^Genetics and Precision Medicine Department, King Abdullah Specialized Children's Hospital, King Abdulaziz Medical City, Ministry of National Guard Health Affairs, Riyadh, Saudi Arabia; ^2^Department of Pediatrics, Faculty of Medicine, Kuwait University, Jabriya, Kuwait; ^3^Department of Clinical Sciences, College of Medicine, University of Sharjah, Sharjah, United Arab Emirates; ^4^Department of Pediatrics, University Hospital Sharjah, Sharjah, United Arab Emirates; ^5^Genetics and Metabolic Department, KidsHeart Medical Center, Abu Dhabi, United Arab Emirates

**Keywords:** WIPI, WD repeat domain, autophagy, neurodevelopment, WDR

## Abstract

WIPI (*W*D-repeat protein *I*nteracting with *P*hospho*I*nositides) are important effectors in autophagy. These proteins bind phosphoinositides and recruit autophagy proteins. In mammals, there are four WIPI proteins: WIPI1, WIPI2, WIPI3 (WDR45B), and WIPI4 (WDR45). These proteins consist of a seven-bladed β-propeller structure. Recently, pathogenic variants in genes encoding these proteins have been recognized to cause human diseases with a predominant neurological phenotype. Defects in *WIPI2* cause a disease characterized mainly by intellectual disability and variable other features while pathogenic variants in *WDR45B* and *WDR45* have been recently reported to cause El-Hattab-Alkuraya syndrome and beta-propeller protein-associated neurodegeneration (BPAN), respectively. Whereas, there is no disease linked to *WIPI1* yet, one study linked it neural tube defects (NTD). In this review, the role of WIPI proteins in autophagy is discussed first, then syndromes related to these proteins are summarized.

## Introduction

Autophagy is a highly conserved process that results in delivery of cellular cargo to the lysosome for degradation and recycling (Parzych and Klionsky, 2014). Three types of autophagy are recognized: microautophagy, macroautophagy, and chaperone-mediated autophagy. Macroautophagy (thereafter referred to as autophagy) is the one best studied of the three types. It is characterized by sequestration of cellular cargo within a double-membraned vesicle (autophagosome) to fuse with lysosomes for degradation and recycling (Parzych and Klionsky, 2014; Klionsky et al., 2021). Autophagy could be nonselective, turning over bulk cytoplasm under starvation conditions, or selective, targeting specific cargoes, such as protein aggregates or damaged organelles (Feng et al., 2014).

There are different physiological roles for autophagy. Autophagy is essential for the maintenance of cellular homeostasis. It is an anti-stress mechanism that is activated in response to various stress stimuli such as hypoxia and starvation (Kroemer et al., 2010). Autophagy has also been implicated in different stages of life cycle in mammals (Wang et al., 2019b; Allen and Baehrecke, 2020), as evidence shows that it is important for maintenance of stem cell function (Dong et al., 2021). Furthermore, autophagy also regulates the response of the immune system (Germic et al., 2019). Given the myriad roles of autophagy in normal physiological, dysregulation of this process has been implicated in a number of diseases, such as cancer, neurodegenerative diseases, and metabolic disorders (Lei and Klionsky, 2021). Congenital disorders of autophagy is now an emerging class of inborn errors of metabolism encompassing different disorders that are mostly neurodegenerative in nature (Ebrahimi-Fakhari et al., 2016). Among these congenital disorders of autophagy are WIPI-related disorders that will be discussed in this review.

## Autophagy and WIPI proteins

In mammals, autophagosome formation is a complex process that consists of several steps starting from initiation and nucleation to lipidation and expansion, and finally to closure and fusion (Vincent et al., 2021). Many autophagy-related proteins (ATG) were first identified by Ohsumi in 1993 (Tsukada and Ohsumi, 1993) in yeast to be involved in these processes through the arrangement into functional complexes. Autophagosome formation is initiated by the assembly of the core autophagy machinery proteins into a multi-component structure called the phagophore assembly site (PAS) (Hollenstein and Kraft, 2020). The initial stage of autophagosome formation results in a double membrane sac called the phagophore. Unc-51-like kinase 1/2 (ULK1/2) complex, which consists of ULK1/2, ATG13, FIP200 and ATG101, is a key autophagic protein complex involved at the initial stage (Dikic and Elazar, 2018). One of the main regulators of autophagy is AMP-activated protein kinase (AMPK) which senses low energy level at the cell and induces autophagy through phosphorylation and inhibition of mammalian target of rapamycin complex 1 (mTORC1) (Rabanal-Ruiz et al., 2017). mTORC1 is a negative regulator of autophagy induction through phosphorylation and inactivation of ULK1/2 and ATG13. mTORC1 also inhibit autophagy through phosphorylation and subsequent degradation of WIPI2 (Wan et al., 2018; Wan and Liu, 2019). AMPK can activate autophagy indirectly through TSC1/2 complex; an intermediate protein complex which also can inhibit mTORC1. TSC1/2 complex activation is regulated through LKB1 (liver kinase B1)-mediated AMPK activation which phosphorylates TSC1/2 (Rabanal-Ruiz et al., 2017; Dikic and Elazar, 2018). Another complex that is essential for initiation of autophagy is VPS34 complex that includes phosphoinositide 3-kinase (PI3K). Colocalization of ULK1/2 and VPS34 complexes at the endoplasmic reticulum (ER) is particularly important for the production of phosphatidylinositol 3-phosphate. Phosphatidylinositol 3-phosphate pool at the ER acts as a signaling molecule to recruit downstream autophagy proteins, triggering next steps in the autophagy process (Mercer et al., 2018).

**Figure 1:**
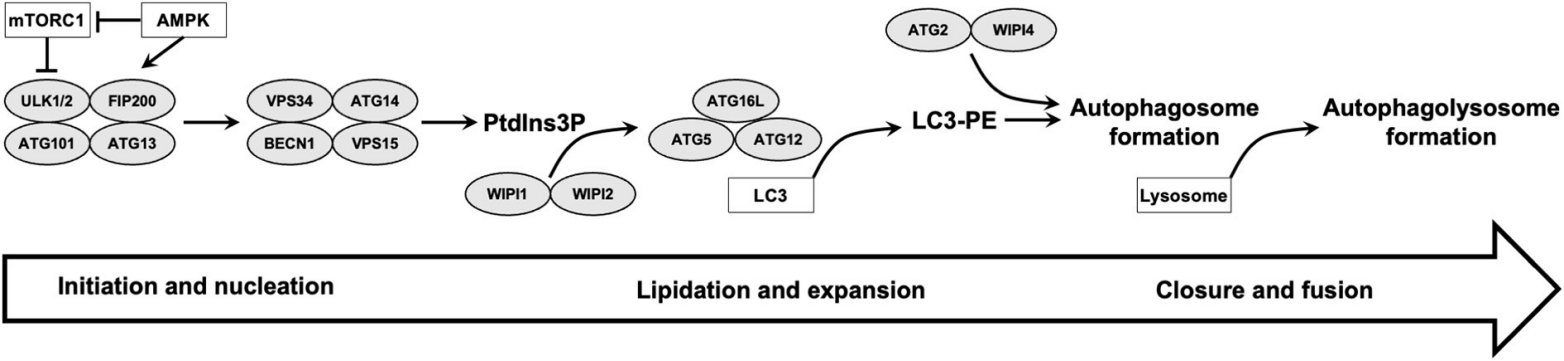
Summary of different steps of autophagy and role of WIPI proteins is illustrated. ULK1/2 and VPS34 complexes are involved in initiation and nucleation steps. The produced phosphatidylinositol 3-phosphate (PtdIns3P) pool recruit downstream autophagy proteins, including WIPI2 which recruits ATG16L complex for LC3 lipidation and autophagosome formation. WIPI4 binds to ATG2 and the ATG2-WIPI4 complex acts as a tethering factor for phagophore expansion. Finally, the expanding autophagosome closes and fuses with lysosomes.

The resulting phagophore from the initial step is then expanded to form the double-membrane autophagosome. Two ubiquitin-like conjugation pathways are essential in this second step: the microtubule-associated protein 1 light chain 3 (LC3) system and the ATG16L1 complex (ATG12–ATG5–ATG16L). These systems are involved in the conjugation of LC3 to phosphatidylethanolamine (LC3-II), a process that is called LC3 lipidation (Proikas-Cezanne et al., 2015; Dikic and Elazar, 2018). ATG2 protein is also involved in phagophore expansion through transfer of lipids from the ER for autophagosome formation (Maeda et al., 2019). Autophagosome closure involves fission of the phagophore membranes and this is regulated by ESCRT (endosomal sorting complexes required for transport) machinary (Jiang et al., 2021). Finally, the expanding autophagosome sequestering cytoplasmic material fuses with endosomes and lysosomes for degradation and the fusion process require SNARE (soluble *N*-ethyl maleimide-sensitive protein (NSF) attachment protein receptor) complexes (Jiang et al., 2021) (Figure 1).

PROPPIN (β-propellers that bind polyphosphoinositides) proteins are effectors of phosphoinositide and they are involved in several steps in autophagy. They bind to phosphoinositide to recruit downstream ATG proteins (Bakula et al., 2017). While there are three PROPPINs in yeast (Atg18, Atg21, and Hsv2), mammals have four members termed WIPI (*W*D-repeat protein *I*nteracting with *P*hospho*I*nositides) proteins: WIPI1, WIPI2, WIPI3 (WDR45B), and WIPI4 (WDR45) (Ren et al., 2020; Vincent et al., 2021). WIPI proteins can be classified into two paralogous subgroups; WIPI1 and WIPI2 in the first group, and WIPI3 (WDR45B) and WIPI4 (WDR45) in the second group (Ji et al., 2019). WIPI proteins contains seven WD40 repeats arranged into seven-bladed β-propeller structure (Liang et al., 2019). Each WD40 repeat (WDR) is a structural motif comprised of approximately 40 amino acids often terminating in a tryptophan-aspartic acid (W-D) dipeptide. Blades 5 and 6 have two distinct phosphatidylinositol-phosphate-binding sites and embedded between them is a highly-conserved signature FRRG (Phe-Arg-Arg-Gly)/LRRG (Leu-Arg-Arg-Gly) motif that plays a major role in phosphatidylinositol-phosphate recognition and autophagy function (Liang et al., 2019; Ren et al., 2020).

WIPI proteins are involved in different stages of autophagy. WIPI1 and WIPI2 are the first two WIPIs to be recruited. WIPI2 interacts with components of ULK1/2 complex and phosphatidylinositol-3-phosphate and this interaction tethers phagophore to the ER (Zhao et al., 2017). Vacuole membrane protein 1 (VMP1), an ER-localized metazoan-specific protein, then facilitates detachment between phagophore and ER for autophagosome formation (Zhao et al., 2017).

WIPI1 and WIPI2 function upstream of the ATG16L1 and LC3 conjugation systems, thereby regulating LC3 conjugation to phosphatidylethanolamine (Polson et al., 2010; Gaugel et al., 2012). WIPI2 bind to phosphatidylinositol 3-phosphate and recruits ATG16L complex for LC3 lipidation and autophagosome formation while WIPI1 supports this function (Dooley et al., 2014). Bansal et al. showed that autophagy receptor optineurin is involved in this step through facilitating the recruitment of the ATG16L1 complex to WIPI2-positive phagophores (Bansal et al., 2018). Interestingly, WIPI2 is a target for autophagy inhibition during mitosis and this is achieved through CUL4-RING ubiquitin ligases mediated polyubiquitination and proteasomal degradation (Lu et al., 2019).

WIPI3 and WIPI4 can act as scaffolds to integrate the LKB1-AMPK-TSC1/2 pathway. WIPI3 has regulatory role in mTORC1 inhibition through interaction with TSC1 (Bakula et al., 2017). Chowdhury et al. showed that WIPI4 binds to ATG2 and the ATG2-WIPI4 complex act as a tethering factor for phagophore expansion *via* the transfer of lipid membranes from the ER and/or the vesicles to the phagophore (Chowdhury et al., 2018). WIPI4 forms a complex with AMPK and ULK1 under fed conditions and this complex is released under starvation to localize together with ATG2 at the phagophore (Bakula et al., 2017). WIPI3 and WIPI4 recognize a peptide sequence from ATG2, termed WIR (WIPI-interacting-region)-motif and mutations in the WIPI3 and WIPI4 peptide-binding sites affect interactions with ATG2 and therefore impair the ATG2-mediated autophagic process (Ren et al., 2020). Ji et al. showed that WDR45B and WDR45 double knockout mice had a more severe autophagy defects and died early suggesting that these two proteins act together in autophagy (Ji et al., 2019).

Beside their major roles in autophagy, WIPI proteins have other functions (Vincent et al., 2021). WIPI1 is also involved in endosome trafficking (Jeffries et al., 2004) and transferrin receptor recycling to the plasma membrane (De Leo et al., 2021). WIPI3 has been implicated in other form of autophagy known as GOMED (Golgi-membrane-associated degradation) that generates autophagic structures from Golgi membranes (Yamaguchi et al., 2020).

## WIPI proteins and human disease

### WIPI1

*WIPI1* is located on chromosome 17q24.2 and it was the first gene to be identified among the WIPI family (Proikas-Cezanne et al., 2015). There is no human disease listed in OMIM that is linked to this gene yet. In a cohort of patients with neural tube defects, Wang et al. identified four patients with rare missense variants in *WIPI1* (Wang et al., 2019a). They showed that knockdown or overexpression of wild type (WT) *WIPI1* in zebrafish caused convergent extension defects thus suggesting a role of WIPI1 in neural tube formation (Wang et al., 2019a). Furthermore, WIPI1 is highly expressed in osteosarcoma cells and it promotes osteosarcoma cell proliferation (Ran et al., 2021). WIPI1 is also upregulated in melanoma and can be used as a tumor marker (D'Arcangelo et al., 2018). Using transcriptomic data, Tzimas *et al*. showed that increased WIPI1 signaling is involved in right ventricular failure (RVH) and silencing of *WIPI1* in aldosterone-stimulated rat cardiac myocytes blunted excessive noncanonical autophagy and mitochondrial oxidative stress, suggesting that WIPI1 could be a potential therapeutic target in RVH (Tzimas et al., 2019).

### WIPI2

*WIPI2* is located on chromosome 7p22.1. WIPI2 is ubiquitously expressed, and it has different isoforms with the main one being WIPI2b. One study showed that aged mice had a striking accumulation of autophagic structures, and overexpression of WIPI2 was sufficient to restore the rate of autophagosome formation in the neurons from these mice (Stavoe and Holzbaur, 2019). Interestingly, Stavoe et al. showed that WIPI2 is dynamically phosphorylated, and this is critical for autophagosome biogenesis. Age-related misregulation of WIPI2 phosphorylation contributes to the age-related decline in neuronal autophagy (Stavoe and Holzbaur, 2019).

*WIPI2* was first reported as a candidate disease-causing gene in a cohort of subjects with unexplained cerebral palsy in an individual with a monoallelic *de novo* predicted-pathogenic variant (McMichael et al., 2015). In 2019, Maddirevula et al. reported one individual with hypotonia, failure to thrive, nystagmus, and elevated transaminases and α-fetoprotein who harbored a homozygous truncating variant in *WIPI2* (Maddirevula et al., 2019). In the same year, Jelani et al. reported a large consanguineous Pakistani family with multiple affected members who presented with a neurodevelopmental disorder characterized by intellectual disability (ID) (Jelani et al., 2019). Detailed clinical information was described for two affected members who had, besides ID, other features including receptive and expressive language delay, short stature, mildly increased thoracic kyphosis, mild bilateral thumb hypoplasia and clinodactyly, wide-based gait, and ECG abnormalities with normal echocardiogram. Brain computed tomography (CT) showed mild, global brain volume loss and prominence of the ventricles. A homozygous missense variant was identified in in *WIPI2* (c.745G>A;pV249M) that segregated with the disease in 10 affected members (Jelani et al., 2019). This missense variant affects a highly conserved region of WIPI2 involved in binding with phosphatidylinositol-phosphate. Functional studies of fibroblasts from affected individuals showed that binding of the mutant protein to ATG16L1 was significantly reduced. Furthermore, starved cells had weakly stained, smaller LC3 puncta and no WIPI2 puncta detectable. Overall, these fibroblasts showed an altered response to starvation and reduced autophagic flux (Jelani et al., 2019).

Recently, Maroofian et al. identified two additional consanguineous families of Egyptian and Saudi origin with two different homozygous missense variants in *WIPI2* (c.551T>G; p.V184G and c.724C>T; p.R242W) (Maroofian et al., 2021). Three individuals from the Egyptian family harboring the V184G missense variant presented with a more severe phenotype compared to the Pakistani family previously-reported in Jelani et al. (2019), as they presented with microcephaly, profound global developmental delay (GDD)/ID, refractory and early onset seizures, dyskinesia, and progressive tetraplegia with joint contractures. Variable dysmorphic features were described including long face with a prominent chin, thick eyebrows, prominent nose, thick alveolar ridge, dental deformities and large ears (Maroofian et al., 2021). The fourth new individual in Maroofian *et al*. from the Saudi family had a milder phenotype with GDD, nystagmus, cone–rod dystrophy, ataxic gait, behavioral concerns with stereotyped movements and self-injurious behaviors, and autistic features. Additional clinical features on the two Pakistani individuals reported by Jelani *et al*. were also provided by Maroofian et al. in which there was no regression or additional features. Furthermore, at the ages of 47 and 50 years, these Pakistani adult patients continued to have moderate ID. Interpretation of the brain magnetic resonance imaging (MRI) of all five individuals with *WIPI2* biallelic variants reported in Maroofian et al. (including the four new individuals and one individual from the previously reported Pakistani family) showed prominent white matter involvement with a global reduction in volume, posterior corpus callosum hypoplasia, abnormal dentate nuclei and hypoplasia of the inferior cerebellar vermis (Maroofian et al., 2021). Functional studies revealed that expression of the Val166Gly mutant protein in *WIPI2* KO HEK293A cells was not able to rescue the deficient LC3 lipidation whereas Arg224Trp mutant increased LC3 lipidation, suggesting a dysregulation of the early steps of the autophagy pathway (Maroofian et al., 2021). In summary, defects in *WIPI2* result in a neurodevelopmental disease (MIM# 18453) characterized by GDD/IDD, reduced brain volume, and variable other features including seizures, skeletal abnormalities, and dysmorphic features (Table 1).

**Table 1:** Lists of WIPI related disorders.

**Gene**	**Disease**	**Main features**	**Inheritance**	**Prevalence**
*WIPI2*	Intellectual developmental disorder with short stature and variable skeletal anomalies (MIM# 18453)	GDD/IDD, reduced brain volume, and variable other features including seizures, skeletal abnormalities, and dysmorphic features.	AR	Three reported families to date
*WIPI3 (WDR45B)*	El-Hattab-Alkuraya syndrome (MIM# 617977)	Profound GDD, early-onset refractory seizures, microcephaly, and progressive spastic quadriplegia	AR	12 reported families to date
*WIPI4 (WDR45)*	Beta-propeller protein-associated neurodegeneration (BPAN) (MIM# 300894)	Biphasic presentation with early childhood onset seizures that decrease over time and GDD with minimal or absent speech. Progressive dementia, parkinsonism, and dystonia in adolescent or early adulthood.	XLR	2–3/million

AR, autosomal recessive; GDD, global developmental delay; intellectual disability; XLR, X-linked recessive.

### WIPI3 (WDR45B)

*WDR45B* is located on chromosome 17q25.3. *WDR45B* knockout mice developed motor deficits and learning and memory defects and their brains showed autophagy substrates accumulation in various brain regions (Ji et al., 2019). WDR45 and WDR45B are specifically required for neural autophagy (Ji et al., 2019).

*WDR45B* was first linked to human disease in 2011. In a cohort of individuals with ID, Najmabadi et al. identified three siblings with ID and microcephaly with a homozygous missense variant in *WDR45B* (Najmabadi et al., 2011). Six years later, an additional individual with GDD and microcephaly with a homozygous nonsense variant in *WDR45B* was identified (Anazi et al., 2017). The first detailed clinical report of *WDR45B*-associated neurodevelopmental disorders was published in 2018 by Suleiman et al. who described six individuals from three unrelated families with a shared overlapping phenotype of profound GDD, early-onset refractory seizures, and progressive spastic quadriplegia (Suleiman et al., 2018). This syndrome is now known as El-Hattab-Alkuraya syndrome (MIM#617977).

Recently, our group published detailed clinical, molecular, and radiological features of 12 new subjects with El-Hattab-Alkuraya syndrome (Almannai et al., 2022). Similar to what was observed in the initial report, the main features of this syndrome in this cohort are GDD, spastic quadriplegia, microcephaly, and early onset and refractory epilepsy (Almannai et al., 2022). Furthermore, neuroradiological findings in this syndrome are homogenous, especially among individuals with loss-of-function variants and therefore four criteria were proposed that could suggest the diagnosis of El-Hattab-Alkuraya syndrome. These include: cerebral atrophy that is disproportionately most prominent in frontal lobes, *ex-vacuo* ventricular dilatation with notable posterior-horn predominance, brainstem atrophy with flattening of belly of the pons, and symmetric under-opercularization. A possible genotype-phenotype correlation was demonstrated as two individuals with a homozygous missense variant had a milder phenotype with less prominent clinical and radiological features compared to the rest of subjects with complete loss-of-function variants (Almannai et al., 2022).

### WIPI4 (WDR45)

*WDR45* is located on chromosome Xp11.23 and is ubiquitously expressed in all tissues. Noda *et al*. showed that *WDR45* is expressed in a developmental stage-dependent manner in mouse brain and it is essential in dendrite development, axon pathfinding and synapse formation (Noda et al., 2021). In 2012, this gene was first linked to human disease when Haak et al. showed different *de novo* pathogenic *WDR45* variants in a group of individuals with neurodegeneration with brain iron accumulation (NIBA) (MIM# 300894) (Haack et al., 2012). These individuals presented with common features including early onset GDD, parkinsonism, and dystonia. They developed dementia by early adulthood. Brain MRI showed iron deposition in the substantia nigra and globus pallidus. A unique feature of this form of NBIA was T1 hyperintensity surrounding a central linear region of signal hypointensity within the substantia nigra and cerebral peduncles. Although *WDR45* is located on X chromosome, males and females had similar phenotype and the authors suggested this could be due to somatic mutations in males and skewed X inactivation in females (Haack et al., 2012). In another study, Saitsu et al. identified five individuals (age 28–51 years) with *de novo* variants in *WDR45* and NBIA. Lower autophagic activity and accumulation of aberrant early autophagic structures were demonstrated in the lymphoblastoid cell lines of the affected subjects (Saitsu et al., 2013). CNS-specific WDR45 knockout mice recapitulated human phenotype with poor motor coordination, and impaired learning and memory (Zhao et al., 2015). They showed extensive axon swelling with numerous axon spheroids, and abnormal autophagy (Zhao et al., 2015).

The disorder is named now beta-propeller protein-associated neurodegeneration (BPAN) (no longer static encephalopathy with neurodegeneration in adulthood) (Gregory et al., 1993). BPAN is a rare disease and most reported cases are females, possibility due to lethality in male hemizygotes (Hayflick et al., 2013). Characteristic clinical features include biphasic presentation with early childhood onset seizures of different types and GDD with minimal or absent speech. While seizure frequency and severity decrease over time, progressive dementia, parkinsonism, and dystonia start to develop in adolescent or early adulthood. Other reported features include behavioral problems, sleep disorders, and ophthalmological abnormalities (Gregory et al., 1993; Hayflick et al., 2013). A consensus clinical management guideline for BPAN was recently published (Wilson et al., 2021). Besides BPAN, other phenotypes associated with variants in *WDR45* include Rett-like syndrome, intellectual disability, developmental and epileptic encephalopathy and West syndrome (Cong et al., 2021).

Pathogenic variants causing BPAN affect two WDR45 residues involved in ATG2 binding suggesting that X-linked NBIA is due to a defect in the function of the WIPI4-ATG2 complex. Moreover, how these variants affect ATG2 binding could affect the severity of the phenotype (Bueno-Arribas et al., 2021). Fibroblasts from two affected individuals with BPAN showed upregulation of toxic iron suggesting alterations in iron homeostasis (Ingrassia et al., 2017). WDR45 deficiency impairs autophagic degradation of transferrin receptor, leading to excessive iron import and accumulation which then promotes ferroptosis (Xiong et al., 2021). Aring et al. generated *WDR45*-knockout SH-SY5Y neuroblastoma cell line using CRISPR-Cas9-mediated genome editing and they showed loss of WDR45 led to defects in ferritinophagy, a form of autophagy that degrades the iron storage protein ferritin (Aring et al., 2022). Furthermore, loss of WDR45 function resulted in mitochondrial iron accumulation and enhanced reactive oxygen species (ROS) production, which could also contribute to neurodegeneration (Aring et al., 2022).

## Conclusion and future directions

In this minireview, we discussed the role of WIPI proteins in autophagy. While WIPI1 and WIPI2 are involved in LC3 conjugation and phagophore expansion; WIPI3 and WIPI4 integrate the LKB1-AMPK-TSC1/2 pathway and they also bind ATG2, mediating phagophore expansion. Given the essential roles of WIPI proteins in different stages of autophagy, it is not surprising that defects in these proteins are associated with human diseases. Three proteins, WIPI2, WDR45B, WDR45, are already associated with Mendelian neurodegenerative diseases. The three disorders share common features including the progressive neurodegenerative nature, GDD/ID, microcephaly, and seizures. These disorders are inherited as autosomal recessive traits, except for BPAN which has X-linked inheritance. Given that three β-propeller proteins are now associated with neurodegenerative diseases, the term BPAN may not be appropriate, and it might better be replaced with X-linked neurodegeneration with brain iron accumulation (X-linked NIBA) to describe *WDR45*-related disorder. Understanding the precise roles of these proteins in autophagy and the availability of animal models could help in identifying potential therapeutic approaches.

## Author contributions

MA: original draft preparation. DM and AE-H: review and editing. AE-H: supervision. All authors have read and agreed to the published version of the manuscript.

## Conflict of interest

The authors declare that the research was conducted in the absence of any commercial or financial relationships that could be construed as a potential conflict of interest.

## Publisher's note

All claims expressed in this article are solely those of the authors and do not necessarily represent those of their affiliated organizations, or those of the publisher, the editors and the reviewers. Any product that may be evaluated in this article, or claim that may be made by its manufacturer, is not guaranteed or endorsed by the publisher.
